# The Scope of Originality

**Published:** 1913-05

**Authors:** Alice G. Edgerly


### The Scope of Originality.

BY ALICE G. EDGERLY.

It has been written, "As a man thinketh, so is he." It may be said as truly that as a writer expresses himself, so is originality in the literary world. There are but few topics that are really new. The scope of the author of today is merely to tell the old ideas with an attempt to disguise them in a dress of novelty. The first comers seized upon everything available, leaving for those who came after the privilege only of rearranging and redecorating. Each has his own mode of expression, and in this alone lies originality.

As an illustration of this, take the time-worn theme of the following limerick:

There was a young lady named Perkins,
Exceedingly fond of green gherkins;
But she ate so much spice,
And other things nice,
That she injured her internal workin's.

The poet Longfellow, pursuing this theme, would have fallen naturally into trocaic tetrameter, producing something like this:

Hear this tale of Mary Perkins,
Giddy, greedy, foolish maiden;
Poor, unwise, misguided woman!
In green gherkins she delighted,
Small and spicy sour pickles,
Ate she of these toothsome dainties,

Morning, noon and dusky evening,
Till digestive apparatus
Suffered from her indiscretion

Bryant's rendition possibly would have been done in this form:

To her who in the pangs of hunger flies
To sour pickles and to spicy things,
She finds a sure disaster, in her youthful hours,
She may eat much and with impunity
But there glides into her later years an indigestion,
That steals away her gladness ere she is aware.

This could reasonably have been Sir Walter Scott's version:

The girl at eve did eat her fill,
Of sour, spicy picalill;
And soon there was foundation laid,
Of future sorrows for the maid,
And when her college course was done,
There were no honors she had won.
The cause was plain; the midnight food,
In which she had indulged, gave rude,
Dyspepsia autocratic power,
And now discomfort is her dower.

Shakespeare would have been more formal:

To eat or not eat; that is the question:
Whether 'tis nobler suffering in one's vitals,
The terrors of an undigested gherkin,
Or to take steps against a world of trouble,
And sign the pledge against them. To sleep,
To sleep all night; and by a sleep to say
We end the nightmare in digestion brings, and aches
That flesh is heir to, 'tis a situation
Devoutly to be wished.

Characteristic of James Whitcomb Riley would be this:

Awf'lest girl to eat green gherkins,
An' spicy things is Mary Perkins,
She'll eat a lot 'n' then some more;
Dyspepsia's knockin' at her door—
An' here's the way she'll look:

Benjamin Franklin Franklin would have approached the subject from an entirely different standpoint:

Pride breakfasted on gherkins, dined on chowchow, and supped with indigestion.

Manufacturers make foods and fools eat them.

It is foolish to lay out money in the purchase of repentance.

Early to bed and early to rise,
Makes a girl healthy, wealthy and wise.

Who dainties love shall dyspeptics prove,
A word to the wise is enough.

Immortal Browning might have rendered it in this style:

Spice!
Faster and more fast,
Did that foolish girl devour the repast,
(A bottle of chowchow bought at the store).
When empty and well scraped it lay—
For not an atom remained on the brim,
Miss Perkins knew that she must pay,
Dyspepsia's tax, not an hour away.

Johnson doubtless would have given advice on eating spiced pickles in Johnsonese resembling the following:

The incompatibility of human discrimination and human foibles is appalling to an ascetic ratiocination. The proclivity to this idiosyncrasy of holding nocturnal symposiums at which a substance. wholly exotic to nature, is consumed to satiety, is, besides being a prodigious prodigality, irrational to a degree, since a physical disorder, inimical to comfort, renders gourmandizing a practice to be avoided.

It is not improbable that Kipling would have produced something like this:

When you've heard of Mary Perkins, when you know of all she did,
When you've learned the grief that she's enduring now,
Will you kindly drop a shiny shilling, in my little lid,
For the doctor's bill that's raising such a row?
She's an absent-minded beggar and her appetite is great
For consuming many spicy little gherkins,
And the doctor's very busy writing on the office slate
"I've gone to make a call on Mary Perkins."

*Holland's Magazine.*

